# Causal effects on low Apgar at 5-min and stillbirth in a malaria maternal–fetal health outcome investigation: a large perinatal surveillance study in the Brazilian Amazon

**DOI:** 10.1186/s12936-021-03981-y

**Published:** 2021-11-25

**Authors:** Julio Abel Seijas-Chávez, Melissa S. Nolan, Mary K. Lynn, Maria José Francalino da Rocha, Muana da Costa Araújo, Fernando Luiz Affonso Fonseca, Gabriel Zorello Laporta

**Affiliations:** 1 Centro Universitário FMABC, Fundação ABC, Santo André, SP Brazil; 2 Hospital da Mulher e da Criança do Juruá (HMCJ), Cruzeiro do Sul, AC Brazil; 3 Laboratory of Vector-Borne and Parasitic Diseases, Arnold School of Public Health, University of South Carolina, Columbia, SC USA; 4 Universidade Federal do Acre (UFAC) – Forest Campus, Cruzeiro do Sul, AC Brazil; 5 Vigilância Entomológica da Secretaria Municipal de Saúde, Cruzeiro do Sul, AC Brazil; 6 Departamento de Farmácia, Universidade Federal de São Paulo (UNIFESP), Diadema, SP Brazil

**Keywords:** Infectious Pregnancy Complications, Maternal–Fetal Medicine, Maternal Health, Newborns, *Plasmodium falciparum* Malaria, *Vivax* Malaria

## Abstract

**Background:**

Malaria elimination in Brazil poses several challenges, including the control of *Plasmodium falciparum* foci and the hidden burden of *Plasmodium vivax* in pregnancy. Maternal malaria and fetal health outcomes were investigated with a perinatal surveillance study in the municipality of Cruzeiro do Sul, Acre state, Brazilian Amazon. The research questions are: what are the causal effects of low birth weight on low Apgar at 5-min and of perinatal anaemia on stillbirth?

**Methods:**

From November 2018 to October 2019, pregnant women of ≥ 22 weeks or puerperal mothers, who delivered at the referral maternity hospital (Juruá Women and Children’s Hospital), were recruited to participate in a malaria surveillance study. Clinical information was obtained from a questionnaire and abstracted from medical reports. Haemoglobin level and presence of malarial parasites were tested by haematology counter and light microscopy, respectively. Low Apgar at 5-min and stillbirth were the outcomes analysed in function of clinical data and epidemiologic risk factors for maternal malaria infection using both a model of additive and independent effects and a causal model with control of confounders and use of mediation.

**Results:**

In total, 202 (7.2%; *N* = 2807) women had malaria during pregnancy. Nearly half of malaria infections during pregnancy (*n* = 94) were *P*. *falciparum*. A total of 27 women (1.03%; *N* = 2632) had perinatal malaria (19 *P*. *vivax* and 8 *P*. *falciparum*). Perinatal anaemia was demonstrated in 1144 women (41.2%; *N* = 2779) and low birth weight occurred in 212 newborns (3.1%; *N* = 2807). A total of 75 newborns (2.7%; *N* = 2807) had low (< 7) Apgar scores at 5-min., and stillbirth occurred in 23 instances (30.7%; *n* = 75). Low birth weight resulted in 7.1 higher odds of low Apgar at 5-min (*OR* = 7.05, 95% CI 3.86–12.88, *p* < 0.001) modulated by living in rural conditions, malaria during pregnancy, perinatal malaria, and perinatal anaemia. Stillbirth was associated with perinatal anaemia (*OR* = 2.56, 95% CI 1.02–6.42, *p* = 0.0444) modulated by living in rural conditions, falciparum malaria during pregnancy, perinatal malaria, and perinatal fever.

**Conclusions:**

While Brazil continues its path towards malaria elimination, the population still faces major structural problems, including substandard living conditions. Here malaria infections on pregnant women were observed having indirect effects on fetal outcomes, contributing to low Apgar at 5-min and stillbirth. Finally, the utility of employing multiple statistical analysis methods to validate consistent trends is vital to ensure optimal public health intervention designs.

**Supplementary Information:**

The online version contains supplementary material available at 10.1186/s12936-021-03981-y.

## Background

Malaria is a tropical disease responsible for significant global mortality caused by *Plasmodium* parasites and transmitted by *Anopheles* mosquitoes. In 2019, 229 million clinical cases occurred worldwide with 409,000 deaths [1]. In the Americas region, Brazil, Colombia and Venezuela accounted for over 86% of the 0.9 million cases registered in 2019 [1]. In 2019, 152,953 indigenous cases were confirmed by parasitological examination of blood smear slides in Brazil. The largest case burden is concentrated in the Brazilian legal Amazon, which covers the states of Amazonas, Pará, Roraima, Rondônia, Amapá, Mato Grosso, Tocantins, Maranhão and Acre, corresponding to 99.9% of the total malaria reported in the country [2]. In Acre state 12,771 new cases of malaria were reported in 2019, 8.4% of the country’s total, with a malaria incidence rate (MIR) of 14.1 per 1,000 persons. Cruzeiro do Sul, the second most populous municipality in the state was responsible for the largest number of cases: 6,080 cases (MIR = 67.7 per 1000) with 4513 (74.2%) *Plasmodium vivax* only, 1538 (25.3%) *Plasmodium falciparum* only, and 29 (0.5%) mixed infections [2], in 2019.

There is evidence that the risk for malaria, both infection and clinical disease, is higher in pregnant women than in non-pregnant women, likely due to the natural immunologic and hormonal changes occurring during pregnancy [3]. In addition, *P*. *falciparum* binds to the placenta in non-immune primigravidae, which can further induce preterm delivery and intrauterine growth retardation [3, 4]. In places of stable and relatively high transmission, adults including pregnant women have acquired immunity against malaria. Despite the immunological tolerance that occurs during pregnancy, parous women can control but not eliminate, malaria infections [3]. In this high-risk group, asymptomatic infections are common, while clinical malaria is relatively rare [3]. A recent epidemiological study in the Amazonian municipality of Cruzeiro do Sul between 2015 and 2016, reports a prevalence of 7.5% (89/1180) of women having malaria parasite’s DNA in peripheral blood at delivery, while only 7.9% (7/89) of them progressed to symptomatic disease after delivery [5]. Furthermore, 83% (74/89) of these women had *P*. *vivax*, which means *P*. *vivax* infections in pregnancy can be subpatent, hidden from routine surveillance [5]. Clinical outcomes of malaria during pregnancy include maternal anaemia, increased maternal mortality, abortions, delayed intrauterine growth, low birth weight, preterm birth, stillbirths, neonatal deaths, and congenital malaria contributing to higher infant morbidity and mortality [5–12].

In Cruzeiro do Sul, the Juruá Women and Children’s Hospital, is a referral maternity hospital for several municipalities in the Juruá River Valley (Marechal Thaumaturgo, Porto Walter, Rodrigues Alves, Mâncio Lima and Guajará) that coupled with Cruzeiro do Sul have an estimated population of 173,810 inhabitants for 2019 [13]. This endemic and target population study area reported 12,763 malaria cases in 2019 (MIR = 73.4 per 1000) with 9336 *P*. *vivax* only (73.1%), 3359 *P*. *falciparum* only (26.4%), and 68 (0.5%) mixed infections [2]. Beginning in 2015 the Brazil’s Ministry of Health recommended standardized health services in this hospital for routine malaria screening during antenatal care**.** However, domestic exposure risk and endemic transmission cycles still pose a threat to further complicate maternal–fetal health.

**Fig. 1:**
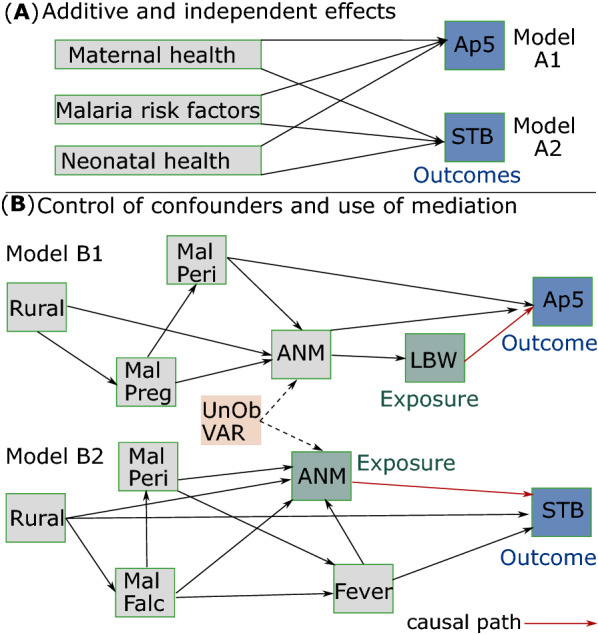
Models to undertake hypothesis testing. **A** Model A1 and Model A2 assume additive and independent effects from component causes to low Apgar at 5-min (Ap5) and stillbirth (STB). **B** An unbiased causal path between exposure, low birth weight at delivery (LBW) or maternal anaemia (ANM) in the perinatal period, and each respective outcome is assumed in Models B1 and B2. The unbiased causal path can be measured after controlling effect from the exposure on the outcome for perinatal malaria (MalPeri), malaria during pregnancy (MalPreg), *P*. *falciparum* during pregnancy (MalFalc), living in rural area (Rural), and perinatal fever (Fever). Mediation is assumed in Models B1 and B2 when ancestors of the exposure modulate the effect on the outcome. The minimal and valid adjustment set for each exposure–outcome relationship is composed by {MalPeri, ANM} in Model B1 and {Rural, Fever} in Model B2. Unobserved variables (UnOb VAR) are factors (e.g., nutritional status, alcoholism, or smoking) that could modulate anaemia, but were not assessed

**Fig. 2:**
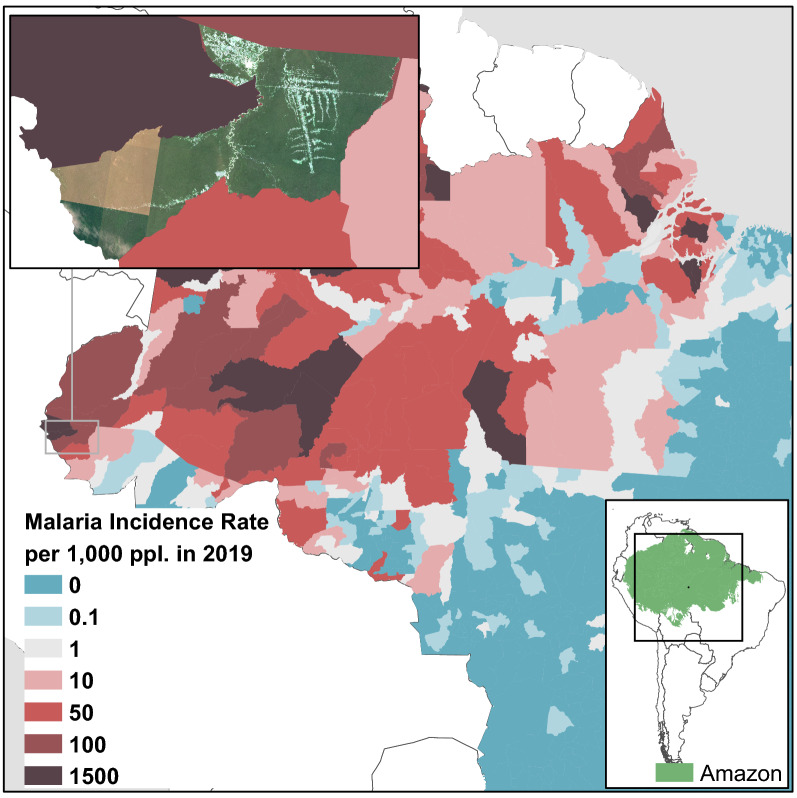
Study location. Acre state Cruzeiro do Sul municipality

This work explores two different statistical approaches to depict the effects of endemic malaria on the health of newborns. The two outcomes of interest are abnormal fetal-to-neonatal transition (Apgar < 7 at 5 min) and stillbirth, and they are assumed to be in function of component causes (Fig. 1A) or caused by exposures modulated by malaria (Fig. 1B). The first analytical approach is based on the sum of additive effects from component causes to each outcome (Model A1, Model A2), assuming that malaria risk factors and maternal and newborn health characteristics are independent (Fig. 1A). The second is based on the control of confounders and the use of mediation to measure unbiased effects of causal paths [14–16]. The outcomes studied caused by newborn low birth weight at delivery (Model B1) and maternal perinatal anaemia (Model B2) are modulated by malaria during pregnancy, perinatal malaria, living in rural area or perinatal fever (Fig. 1B). The research questions are as follows: (1) assuming independent and additive effects, what are the associations between various explanatory variables and the odds of the two outcomes of interest? (Fig. 1A); (2) what is the causal effect of low birth weight on low Apgar at 5-min? (Fig. 1B, Model B1); and (3) what is the causal effect of perinatal anaemia on stillbirth? (Fig. 1B, Model B2). The main goal here is to assess both the proximal and distal causal effects to the outcomes. This is a large hospital-based epidemiological study with a sample of 2807 pregnant women.

## Methods

### Study location

This study took place in Cruzeiro do Sul municipality (Fig. 2), an endemic malaria county in Acre state (Fig. 2). The Juruá Women and Children’s Hospital is an institution maintained by the State Health Department (SESACRE), a member of the Unified Health System (SUS). The hospital unit has a special neonatal ward, including an intensive care unit with multidisciplinary teams, a delivery centre, where vaginal and caesarean deliveries are assisted by obstetricians and nurses, and facilities for outpatient, inpatient, and urgent and emergency assistance in obstetrics and gynaecology; it is equipped with 86 beds, of which 32 correspond to joint accommodation in clinical and surgical obstetrics. The Hospital is a reference for all municipalities in the Juruá River Valley and has auxiliary services for diagnostic support such as biochemistry laboratory, x-rays, electrocardiogram, ultrasonography, and mammography.

The number of extra-hospital deliveries in the region is minimal as it was only 1.4% (42/3090) in the study period, November-2018 to October-2019 [17]. Mothers who live in rural areas and in distant neighbourhoods usually stay at their relatives’ or friends’ homes in the urban area at the first signs of the onset of labour. Even when this is not possible, mothers can be admitted in the Hospital in a latent stage of childbirth.

### Recruitment and procedures

**Fig. 3:**
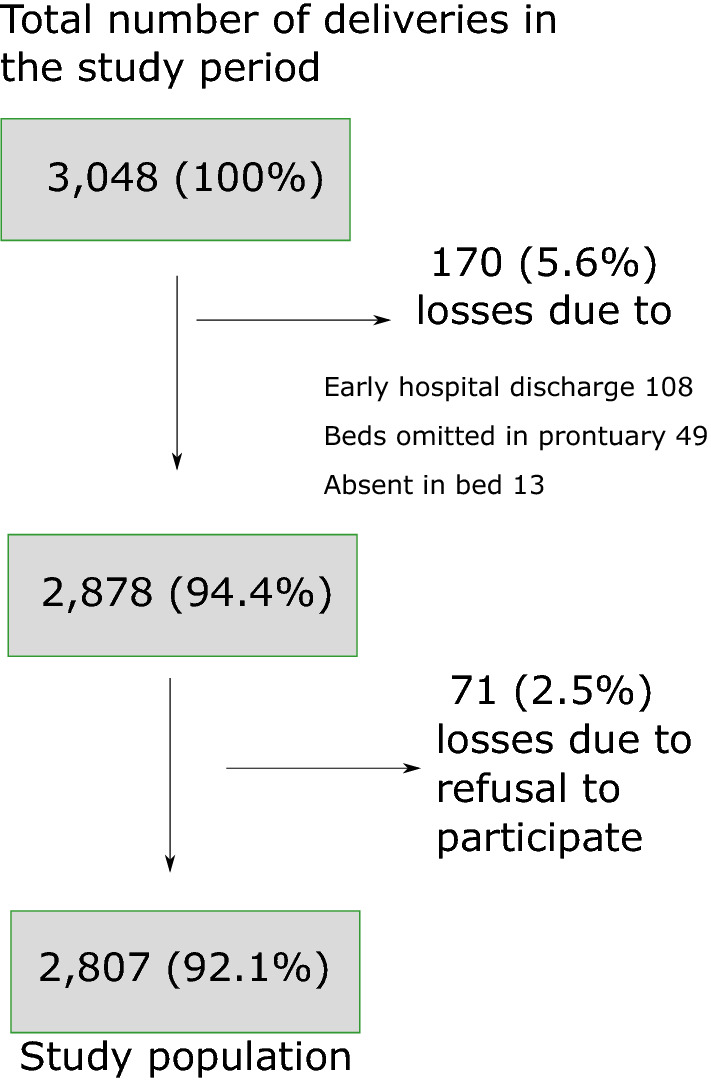
Study population. The final sample population for this study

Pregnant women of ≥ 22 weeks at labour or puerperal mothers (women within 3 days of labour and delivery), who delivered at the Hospital between November 1, 2018 and October 31, 2019 were invited to participate in the research under informed consent signed by the pregnant or puerperal woman, and her legal representative if under the age of consent (< 18 years). Only those not interested in participating in the surveillance research were excluded. A total of *N* = 2807 pregnant women were selected for this study (Fig. 3).

The research tool used in this study is in Additional file 1. At the time of admission to the Hospital, pregnant women in labour or puerperal women of up to 3 days post-delivery, responded to a questionnaire to identify current or prior determinants associated with malaria infection. This questionnaire was based on previous studies about malaria in pregnancy [5, 7, 18]. Further information regarding the period of antenatal care and newborn health was retrieved from patient medical charts. Mother’s blood sample was obtained to estimate haemoglobin (Hgb) levels by automated haematology analyser Counter 19 (Wiener-Lab, Rosario, Argentina), and blood smear conducted to examine malarial parasites by light microscopy Primo Star (Zeiss, Oberkochen, Germany). Treatment of malaria in pregnant women varied per type of *Plasmodium*: (1) *P*. *vivax*, chloroquine 150 mg and (2) *P*. *falciparum* or mixed infection, artemether 20 mg + lumefantrine 120 mg or artesunate 100 mg + mefloquine 200 mg; but primaquine cannot be used [19]. For the control of anaemia among pregnant women (≥ 20 weeks), it was prescribed iron 40 mg and folic acid 400 μg daily. If they had a diagnosis of anaemia, it was prescribed ferrous sulphate 120–240 mg daily [20].

### Data tabulation and variables

Data was tabulated in Epi Info™ v. 7.2. and transformed into categorical and dichotomous variables in *R* programming environment v. 4.0.4. These variables were assumed to follow a Binomial distribution. Variable categorization was based on the following definitions:

### Mothers

1. Malaria in pregnancy: mother diagnosed with any *Plasmodium* during pregnancy.
2. *P*. *falciparum* malaria during pregnancy: mother diagnosed with *P*. *falciparum* or mixed infection (*P*. *falciparum* + *P*. *vivax*) during pregnancy.
3. Rural residence: opposing to a residence within the outskirts of a city, it is a residence in an undeveloped area, where inhabitants engage in activities of agriculture and livestock, extractivism, rural tourism, forestry, or environmental conservation.
4. Perinatal anaemia: mother diagnosed with haemoglobins below 11 g/dL during labour or up to 72 h after delivery.
5. Perinatal malaria: mother diagnosed with perinatal malaria (with any *Plasmodium*) during labour or up to 72 h after delivery.
6. Perinatal fever: mother had fever in the last week prior to the delivery.

### Newborns

7. Delivery: born alive or stillborn after the 5th month of pregnancy (22 weeks or more).
8. Preterm: born alive or stillborn between 22 weeks and 0 days and 36 weeks and 6 days.
9. Low birth weight: born weighing less than 2500 g.
10. Abnormal fetal-to-neonatal transition: newborns with Apgar less than seven at 5 min.

Study variables are shown in Table 1. If there were missing data there for any of the variables (Table 1), the respective individuals were dropped from analysis. Women received haemoglobin blood test either before or after delivery (but never in both periods). One third of the women had haemoglobin blood testing taken after delivery (Table 1). Their haemoglobin levels were significantly lower than those from mothers who had testing taken before delivery. Haemoglobin blood test timing is thus controlling perinatal anaemia in data analysis.

**Table 1:** Absolute (*n*) and relative (%) frequencies of categories in variables under study

Variables studied	Categories	*n* (%)
Outcomes		
Low Apgar at 5-min	Score < 7	75 (2.7)
	Score ≥ 7	2732 (97.3)
Stillbirth	Yes	23 (0.8)
	No	2784 (99.2)
Maternal pregnancy and health characteristics		
Advanced age or young	> 35 years or < 15 years	298 (10.6)
	15–35 years	2509 (89.4)
Number of previous deliveries	1–4 deliveries	1443 (51.4)
	Otherwise	1364 (48.6)
Attended ≥ 6 obstetrics consults during pregnancy	≥ 6 consults	2036 (72.8)
	Otherwise	760 (27.2)
	Missing data	11
Diagnosed with perinatal anaemia	Hgb < 11 g/dL	1144 (41.2)
	Otherwise	1635 (58.8)
	Missing data	28
Presented with perinatal fever	Yes	142 (5.1)
	No	2655 (94.9)
	Missing data	10
Diagnosed with malaria prior to current pregnancy	Yes	1265 (45.1)
	No	1539 (54.9)
	Missing data	3
Diagnosed with malaria during pregnancy	*P*. *vivax* only	108 (3.85)
	*P*. *falciparum* or mixed (falc + viv)	94 (3.35)
	No malaria	2605 (92.8)
Diagnosed with perinatal malaria	Yes	27 (1)
	No malaria	2605 (99)
	Missing data	175
No malaria testing during pregnancy	Testing not done	564 (20.1)
	Testing done	2243 (79.9)
Maternal malaria risk factors		
Resides in Cruzeiro do Sul	Cruzeiro do Sul	1578 (56.2)
	Otherwise	1229 (43.8)
Resides in rural area	Rural	1522 (54.5)
	Urban	1270 (45. 5)
	Missing data	15
Illiterate or has secondary education as highest level	Illiterate or secondary	1072 (38.2)
	High school or college	1734 (61.8)
	Missing data	1
Never used repellent in pregnancy	Not used	684 (24.4)
	Used in any trimester	2123 (75.6)
Never used a bed net during pregnancy	Not used	1078 (38.4)
	Used in any trimester	1779 (61.6)
No air conditioning unit in home	No air conditioning	2333 (83.1)
	With air conditioning	474 (16.9)
Newborn health at delivery		
Born prematurely (in gestational age)	< 37 weeks	203 (7.3)
	≥ 37 weeks	2570 (92.7)
Low birth weight at delivery (g)	< 2500 g	212 (7.5)
	≥ 2500 g	2595 (92.5)
Low head circumference (cm)	< 33 cm	342 (12.2)
	≥ 33 cm	2456 (87.8)
Control variable		
Haemoglobin blood test timing	Before delivery	1795 (64.8)
	After delivery	977 (35.2)
	Missing data	35

### Model A1 and Model A2

Binomial logistic regressions were applied, as in Hosmer et al. [21], to associate low Apgar at 5-min (score < 7 score) (Model A1) and stillbirth (Model A2) with maternal pregnancy and health characteristics, maternal malaria risk factors, and newborn health at delivery (Fig. 1A), adjusted by haemoglobin blood test timing (Table 1), as follows.

$$Prob \left( {y = 1} \right) = \frac{1}{{1 + e^{{ - \left( {\beta_{0} + X_{n} \beta_{n} } \right)}} }},$$

where *y* = outcomes and *X*_*n*_*β*_*n*_ = additive and independent effects from component causes.

The strength and statistical significance of association was calculated with the exponential value of *β*_n_ to estimate the odds ratio (*OR*) of *X*_n_ in relation to *Prob* (*y* = 1).

$$OR\left( {X_{n} } \right) = \exp \left( {\beta_{n} } \right).$$

The following null hypothesis (H_0_: *OR* = 1) was tested with its alternative (H_a_: *OR* ≠ 1) considering 0.05 (type-I error or *α*) significance level and (1 − α) % confidence intervals. *OR* > 1 meant reciprocal association between response and explanatory or control variables, whereas *OR* < 1 meant that this relationship was non-reciprocal. Lastly, *OR* = 1 meant no association (null effect). Using a stepwise forward approach, statistically significant variables in the univariable logistic regression (*p* < 0.05) were selected to the multivariable logistic regression.

### Model B1 and Model B2

A Directed Acyclic Graph (DAG) approach, as in [22], was used to evaluate consistency of each proposed DAG (Model B1 and Model B2) with the study’s dataset (Table 1) and to analyse adjustment sets of valid DAGs per exposure-outcome relationship. Following, the testing of structural equation models (SEM) with binomial errors [23] was employed to measure unbiased causal estimates between low birth weight at delivery and abnormal fetal-to-neonatal transition (Model B1) and maternal perinatal anaemia and stillbirth (Model B2), considering modulation from maternal malaria risk factors (Fig. 1B), supported by [24–28].

In brief, the piecewise structural equation modelling approach was applied to evaluate each of the corresponding binomial logistic regressions in the network of nested models in order to generate inferences about the entire structure [23].

Goodness-of-fit was obtained by testing the set of specified paths with the single Fisher’s C statistic:

$$C = - 2\mathop \sum \limits_{i = 1}^{k} \ln \left( {p_{i} } \right),$$

where $C$ follows a $\chi^{2}$ distribution with 2 $k$ degrees of freedom, with $k$ being the number of specified paths in the structural equation model, and $p_{i}$ = *p*-values from each test combined. If the Fisher’s C statistic null hypothesis was accepted, there were not unspecified paths that could have been included but were omitted (*α* = 0.05). Lastly, it was calculated the *OR* from each estimated coefficient (*β*) as previously, i.e., $OR\left( {X_{n} } \right) = {\text{exp}}\left( {\beta_{n} } \right)$, to test the strength and direction of each specified path, by considering 95% confidence intervals ($\exp \left( {\beta_{n} \pm 1.96 SE_{\beta } } \right)$, *SE* = standard error).

Because 564 (20.1%) of mothers were not tested for malaria during pregnancy, the estimates of association were recalculated excluding these mothers from analyses. Model B1 and Model B2 were re-analysed with mothers (*n* = 2243) who underwent malaria testing during pregnancy. Adjustment sets were maintained as in the previous analyses.

### Software and programming resources

All analyses were performed in *R* programming environment v. 4.0.4 with specific packages *epiDisplay* v. 3.5.0.1, for the calculation of *OR* and 95% CI, *dagitty* v. 0.3–1 [22], for the depiction of consistent and valid DAGs, and *piecewiseSEM* v. 2.1.2 [23], for the modelling of structural equations.

## Results

### Research question 1: assuming independent and additive effects, what are the associations between various explanatory variables and the odds of the two outcomes of interest?

A total of 75 (2.7%) out of 2807 newborns had a low Apgar at 5-min (Model A1 in Fig. 1A). Table 2 shows associations between these newborns (*n* = 75) and those who had normal Apgar at 5-min (*n* = 2732) in relation to maternal characteristics, malaria risk factors, and newborn health. Mothers (*n* = 1144; 41.2%) diagnosed with perinatal anaemia (Hgb < 11 g/dL) were associated with newborns having low Apgar at 5-min in the univariable logistic regression (*OR* = 1.85; 95% CI = 1.17–2.94; *p* = 0.009). Multivariable logistic regression showed that mothers who presented with perinatal fever have higher odds of having a newborn with low Apgar at 5-min (*OR* = 3.47; 95% CI = 1.65–7.29; *p* = 0.001). Newborns who were born prematurely (*OR* = 3.18; 95% CI = 1.52–6.65; *p* = 0.002) and presented at delivery with a low birth weight (*OR* = 3.67; 95% CI = 1.6–8.39; *p* = 0.002) or a low head circumference (*OR* = 2.15; 95% CI = 1.06–4.39; *p* = 0.035) have higher odds of showing low Apgar at 5-min (Table 2).

**Table 2:** Measurement of the associations with low Apgar at 5-min according to maternal characteristics, malaria risk factors, and newborn health (Model A1)

	Univariable logistic regression			Multivariable logistic regression		
	*p*	*OR*	95% CI	*p*	*OR*	95% CI
Outcome						
Low Apgar at 5-min (score < 7)						
Maternal pregnancy and health characteristics						
Advanced age (> 35 years) or young (< 15 years)	0.99	1.01	0.48–2.11			
Number of previous deliveries (n = 1 to 4)	0.55	1.15	0.73–1.82			
Attended ≥ 6 obstetrics consults during pregnancy	< 0.001	0.21	0.13–0.34	0.003	0.41	0.23–0.74
Diagnosed with perinatal anaemia (Hgb < 11 g/dL)	0.009	1.85	1.17–2.94	0.294	1.34	0.78–2.3
Presented with perinatal fever	< 0.001	4.21	2.26–7.86	= 0.001	3.47	1.65–7.29
Diagnosed with malaria prior to current pregnancy	0.78	1.07	0.67–1.69			
Diagnosed with malaria during pregnancy	0.79	1.13	0.48–2.62			
Diagnosed with perinatal malaria	0.13	3.13	0.73–13.5			
No malaria testing during pregnancy	0.77	1.1	0.62–1.91			
Maternal malaria risk factors						
Resides in Cruzeiro do Sul	0.09	1.48	0.94–2.35			
Resides in rural area	0.46	1.19	0.75–1.9			
Illiterate or has secondary education as highest level	0.007	1.88	1.19–2.98	0.087	1.62	0.93–2.81
Never used repellent in pregnancy	0.311	1.3	0.78–2.15			
Never used a bed net during pregnancy	0.165	0.7	0.43–1.16			
No air conditioning unit in home	0.258	1.5	0.74–3.03			
Newborn health at delivery						
Born prematurely (< 37 weeks gestational age)	< 0.001	13.41	8.2–21.8	0.002	3.18	1.52–6.65
Low birth weight at delivery (< 2500 g)	< 0.001	20.4	12.6–33.1	0.002	3.67	1.6–8.39
Low head circumference (< 33 cm)	< 0.001	9.2	5.6–15.1	0.035	2.15	1.06–4.39
Control variable						
Haemoglobin blood test timing	0.725	0.92	0.56–1.49	0.997	0.999	0.567–1.76

Out of all deliveries (*N* = 2807) 23 stillbirths (0.8%) were observed (Model A2 in Fig. 1A). Table 3 shows associations between these stillbirths (*n* = 23) and those who were born alive at delivery (*n* = 2784) in relation to maternal characteristics, malaria risk factors, and newborn health. Mothers diagnosed with perinatal anaemia were associated with stillbirths in the univariable logistic regression (*OR* = 3.12; 95% CI = 1.27–7.68; *p* = 0.013). In the multivariable logistic regression mother who presented with perinatal fever have higher odds of having a stillbirth at delivery (*OR* = 16.94; 95% CI = 4.74–60.49; *p* < 0.001) (Table 3).

**Table 3:** Measurement of the associations with stillbirths according to maternal characteristics, malaria risk factors, and newborn health (Model A2)

	Univariable logistic regression			Multivariable logistic regression		
	*p*	*OR*	95% CI	*p*	*OR*	95% CI
Outcome						
Stillbirth						
Maternal pregnancy and health characteristics						
Advanced age (> 35 years) or young (< 15 years)	0.016	3.2	1.24–8.24	0.11	2.9	0.8–10.6
Number of previous deliveries (n = 1 to 4)	0.258	0.6	0.25–1.45			
Attended ≥ 6 obstetrics consults during pregnancy	< 0.001	0.1	0.04–0.3	0.041	0.19	0.04–0.93
Diagnosed with perinatal anaemia (Hgb < 11 g/dL)	0.013	3.12	1.27–7.68	0.529	1.48	0.43–5.06
Presented with perinatal fever	< 0.001	13.3	5.5–32.3	< 0.001	16.94	4.74–60.49
Diagnosed with malaria prior to current pregnancy	0.15	1.9	0.8–4.4			
Diagnosed with malaria during pregnancy	0.17	2.8	0.7–12.2			
Diagnosed with perinatal malaria	0.99	0	0, Inf			
No malaria testing during pregnancy	0.77	0.85	0.3–2.5			
Maternal malaria risk factors						
Resides in Cruzeiro do Sul	0.026	2.78	1.1–6.8	0.682	1.3	0.38–4.48
Resides in rural area	0.359	1.5	0.6–3.6			
Illiterate or has secondary education as highest level	0.046	2.39	1.02–5.61	0.477	1.57	0.45–5.49
Never used repellent in pregnancy	0.207	1.75	0.73–4.2			
Never used a bed net during pregnancy	0.517	0.74	0.3–1.8			
No air conditioning unit in home	0.326	2.1	0.5–8.9			
Newborn health at delivery						
Born prematurely (< 37 weeks gestational age)	< 0.001	18.1	7.5–43.4	0.302	2.26	0.48–10.61
Low birth weight at delivery (< 2500 g)	< 0.001	60.6	20.3–181	0.142	4.37	0.61–31.3
Low head circumference (< 33 cm)	< 0.001	20.3	6.4–64.2	0.117	3.99	0.71–22.54
Control variable						
Haemoglobin blood test timing	0.324	1.53	0.66–3.55	0.072	3.04	0.91–10.2

### Research questions 2 and 3: what are the causal effects of low birth weight on low Apgar at 5-min and of perinatal anaemia on stillbirth?

**Fig. 4:**
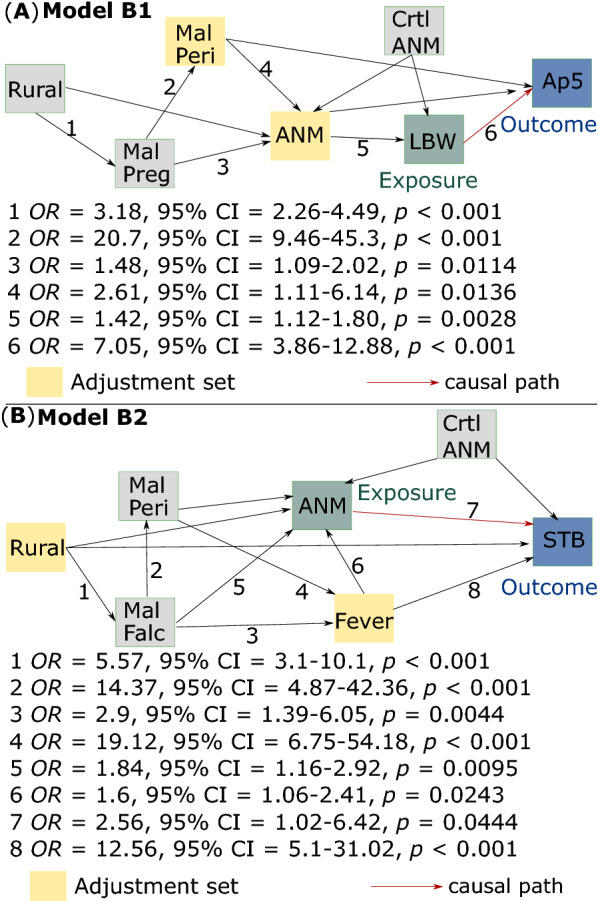
Structural equation modelling. **A** Model B1 and **B** Model B2 were tested by nested binomial logistic regression models with estimation of *OR* and 95% CI. *Rural* mother resides in rural area, *MalPreg* mother diagnosed with malaria during pregnancy, *MalPeri* mother diagnosed with perinatal malaria, *MalFalc* mother diagnosed with *P*. *falciparum* or mixed infections during pregnancy, *Fever* mother presented with perinatal fever, *ANM* mother diagnosed with perinatal anaemia, *Crtl ANM* haemoglobin blood test timing (control variable), *LBW* low birth weight at delivery, *Ap5* low Apgar at 5-min, *STB* stillbirth

No support for a missing link between the causal framework of the proposed DAGs (Fig. 1B) and the study’s dataset (Table 1) was observed, and it was thus assumed the proposed causal frameworks (Models B1 and B2) as consistent. In addition, the same adjustment sets for the exposure-outcome relationship in all equivalent DAGs per model were observed. In Model B1, maternal perinatal malaria and anaemia can adjust the causal path between low birth weight at delivery and low Apgar at 5-min in all equivalent DAGs. The causal path between maternal perinatal anaemia and stillbirth is adjusted in Model B2 by rural residency and perinatal fever in all equivalent DAGs. In other words, these adjustment sets can modulate the exposure-outcome relationship. Figure 4A, B show estimations of *OR* and 95% CI from the testing with SEM. Paths with statistically significant (*p* < 0.05) estimations are shown (Fig. 4A, B).

Model B1 (Fig. 4A) shows that newborns who had low birth weight at delivery have higher odds of low Apgar at 5-min (*OR* = 7.05; 95% CI = 3.86–12.88, *p* < 0.001). These newborns have higher odds of having mothers diagnosed with perinatal anaemia (*OR* = 1.42; 95% CI = 1.12–1.8, *p* = 0.0028). These mothers in turn have higher odds of having experienced both perinatal malaria (*OR* = 2.61; 95% CI = 1.11–6.14, *p* = 0.0136) and malaria during pregnancy (*OR* = 1.48; 95% CI = 1.09–2.02, *p* = 0.0114). Lastly, mothers diagnosed with malaria during pregnancy were 3 times more likely to have residency in rural areas than mothers who lived in the city (*OR* = 3.18; 95% CI = 2.26–4.49, *p* < 0.001). There were no unspecified paths (missing links) in Model B1 (Fig. 4A) (Fisher’s C = 11.886; *p* = 0.854; *d*.*f*. = 18).

In Model B2 (Fig. 4B) stillbirths was associated with higher odds in mothers who had perinatal anaemia (*OR* = 2.56; 95% CI = 1.02–6.42, *p* = 0.0444) and in mothers who presented perinatal fever (*OR* = 12.56; 95% CI = 5.1–31.02, *p* < 0.001). Mothers diagnosed with *P*. *falciparum* or mixed infections during pregnancy had higher odds of presenting perinatal anaemia (*OR* = 1.84; 95% CI = 1.16–2.92, *p* = 0.0095) and perinatal fever (*OR* = 2.9; 95% CI = 1.39–6.05, *p* = 0.0044). And mothers who reside in rural area had higher odds of diagnosis with *P*. *falciparum* or mixed infections during pregnancy (*OR* = 5.57; 95% CI = 3.1–10.1, *p* < 0.001). Model B2 has no missing links (Fisher’s C = 9.31; *p* = 0.811; *d*.*f*. = 14) (Fig. 4B).

Results from an alternative analysis of Model B1 and Model B2 with mothers (*n* = 2243) who underwent malaria testing during pregnancy are shown in Additional file 2. In this analysis both causation models are consistent as they do not retain unspecified paths. Although variations on the strength of *OR* values were observed, overall results were not qualitatively different. The main issue with this analysis is the loss of statistical power due to its 20.1% smaller sample size in comparison with the main analysis here (Fig. 4).

## Discussion

Prevalence of malaria during pregnancy within the studied population was 7.2% (*N* = 202/2807). These results were consistent with previous studies carried out in the same maternity hospital (Women and Children’s Hospital of Juruá Valley) which found prevalence of gestational malaria in the last two decades between 8 and 8.9% [5, 7, 18]. These results are also consistent with gestational malaria prevalence of 6.1% found in the neighbouring municipality of Manaus, Amazonas [29], but differs to those in extra-Amazonian localities (1.5%) [30] or in Africa (41.2%) [31]. Here prevalence of maternal malaria in the study locality was mainly caused by the environment and socioeconomic settings in which mothers and babies live. This initial link may contribute to events such as mothers’ perinatal malaria and anaemia, which can be associated with lower Apgar scores and stillbirth.

The incidence of malaria in the study county (Cruzeiro do Sul) remains unabated, mainly due to deforestation and low socioeconomic status in rural areas [32]. Man-made vector breeding sites including the creation of dams for commercial aquaculture, are among the primary factors leading to increased incidence of disease [33]. This study found nearly half of maternal malaria cases corresponded to *P*. *falciparum* (46.5%; 94/202). This finding is higher than prior studies showing *P*. *falciparum* malaria between 25.4% (31/122) [5] and 36.1% (463/1283) [7] at the study site and 15.5–32.3% in Manaus [25, 29, 34, 35]. It is possible that the higher *P*. *falciparum* prevalence (46.5%) in maternal malaria cases in this study (94/202) may be in part due to the light microscopy limitations in detecting submicroscopic malaria, which is more common among *P*. *vivax* infections [5].

The perinatal malaria prevalence found here was 1.1% (27/2632) by light microscopy, which is similar to that of 0.7% (11/1673) obtained by [18] in the same maternity hospital 10 years ago. The use of PCR-based methods for detecting malaria infection may improve diagnostic capacity compared to microscopy based methods [8, 33]. In the same maternity hospital, Pincelli and colleagues found a much higher perinatal malaria of 7.5% (89/1180) after employment of PCR to diagnose submicroscopic malaria [5]. This hidden burden of maternal malaria due to submicroscopic infections are also observed in Manaus [36] and constitute a challenge to malaria elimination in Brazil—as undiagnosed and untreated asymptomatic reservoirs can perpetuate disease transmission.

**Fig. 5:**
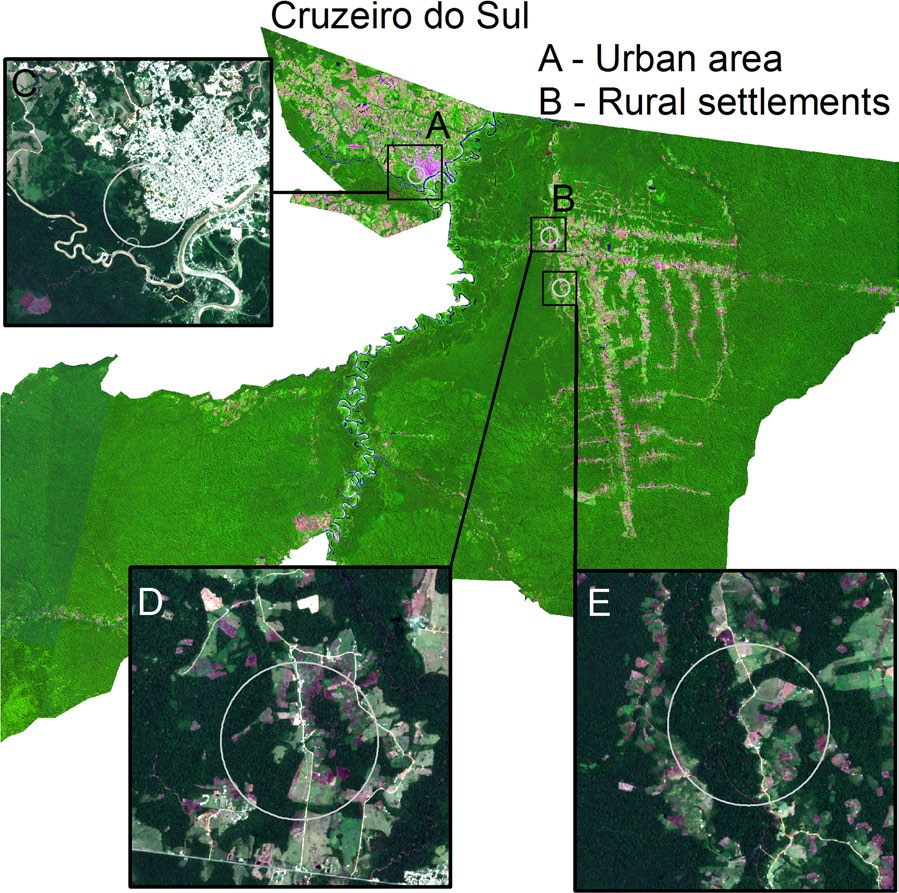
Malaria landscape in Cruzeiro do Sul. **A** Urban area. **B** Rural settlements. **C** Cohab district. **D** PDS Jamil Jereissati—Ramal do José Alves. **E** PDS Jamil Jereissati—Ramal do Caraca (Source: Laporta et al. [40])

Living in the rural area of Cruzeiro do Sul was a consistent yet indirect predictor to maternal–fetal health outcomes found in this study. Prior studies showed that rural living conditions are important maternal malaria determinants [5, 18]. Entomological studies showed occurrence of *P*. *falciparum*-infected anophelines from rural Cruzeiro do Sul [37–41]. Laporta et al. [40] showed that urban and/or peri-urban malaria is mainly caused by *P*. *vivax*-infected anophelines (Fig. 5A), whereas rural settlements have both *P*. *vivax*- and *P*. *falciparum*-infected anophelines (Fig. 5B). *Plasmodium vivax*-infection rate in anophelines is 24% (31/130) in the urban/peri-urban Cohab district (Fig. 5C) and 1.4% (3/210) in rural settlements (Fig. 5D, E) [40]. *Plasmodium falciparum*-infection rate in anophelines is 2.6% (4/153) in the rural settlement Ramal do Caraca (Fig. 5E) [40]. This adds further complexity to interpretating the study findings in that: (1) urban/peri-urban sites have higher *Plasmodium*-infection rate (24% vs. 1.4%) than rural settlements (Fig. 5), but (2) *P*. *falciparum*-infection rate (2.6%) is only present in rural settlements (Fig. 5E) [40]. Having rural living as a strong predictor of maternal–fetal health outcomes can have therefore three straightforward and non-mutually exclusive interpretations: (1) lower access to malaria commodities (diagnosis and treatment) in rural settlements, (2) higher rate of undiagnosed submicroscopic *P*. *vivax* infections which frequently occur in urban/peri-urban sites, and/or (3) higher parasitism and clinical effects from *P*. *falciparum* infections which are only present in rural settings.

Nearly 60% of the surveyed mothers used insecticide treated bed nets (1729/2807) and chemical repellent (2123/2807) in the three trimesters of pregnancy. In 2019 52% of pregnant women in malaria-endemic regions in the world slept under the protection of an insecticide-treated mosquito bed net [1]. However, the exophilic behaviour and bimodal bite pattern of the *Anopheles* (*Nyssorhynchus*) *darlingi*, the main malaria vector, limits the impact that insecticide-treated mosquito bed nets could have on malaria transmission [33]. Here no conclusive protection of mothers using insecticide-treated bed nets against *P*. *falciparum* infection was found. This further shows that mother’s rural residency and *P*. *falciparum* exposure may lead to recurrence of infection with this malarial parasite, independently of the use of anti-mosquito interventions. This escape effect of anophelines from mosquito intervention was proposed in a mathematical modelling study [42]. According to the entomological studies carried out in the study region [38–41, 43], peak *Plasmodium*-infected mosquito biting is skewed towards the night hours from 00:00 to 03:00 when the density of mosquitoes is lowest and therefore higher biting tolerance occurs in human hosts [43].

Mothers having anaemia during labour or up to 72 h after delivery comprised 41.2% of the study sample (1144/2779). The odds of perinatal anaemia are 2.61 times higher (95% CI 1.11–6.14; *p* = 0.0136) if the mother was diagnosed with perinatal malaria (Fig. 4A). The clinical effect of anaemia from malaria infection on pregnancy was related to *P*. *falciparum* infections. The odds of perinatal anaemia were 1.84 times greater (95% CI 1.16–2.92; *p* = 0.0095) if the mother had *P*. *falciparum* infection during pregnancy (Fig. 4B). In Manaus, a multicentre study showed that pregnant women with *P*. *falciparum* had an increased risk for anaemia [36]. Placental malaria, which occurs very frequently in *P*. *falciparum* infection, commonly leads to severe maternal anaemia and consequently low birth weight, especially among primiparous women [8].

It is noteworthy that several studies have shown that *P*. *vivax* and/or *P*. *falciparum* malaria are specifically associated with mother’s anaemia [6, 8]. The successive loss of infected red blood cells, the lysis of the non-infected red blood cells in the circulation and the decrease in the production of red blood cells are known physiological mechanisms underlying this association [4, 44]. In 2009, at the studied maternity hospital, maternal anaemia was the most important effect from malaria infections during pregnancy of 1480 mothers [18]. Another study in the same hospital, between July-2015 and June-2016, observed that a single episode of *P*. *vivax* infection significantly decreased maternal haemoglobin and that repeated infections in the same mother can worsen outcomes (severe anaemia) [5].

Maternal anaemia was here associated with significantly greater odds of having low birth weight (*OR* = 1.42; 95% CI 1.12–1.80; *p* = 0.0028). This association is consistent with the literature [31, 44, 45]. In a peri-urban area of Manaus 2008–2011 a significant association of low birth weight was found with *P*. *falciparum* detected by light microscopy [36]. Low birth weight is responsible for significant infant mortality [8]. Up to 100,000 infant deaths per year in Africa are attributable to low birth weight due to maternal infection with *P*. *falciparum* [31]. In addition, newborns who had low birth weight were more likely to be preterm (*OR* = 35.5; 95% CI 25.11–50.22; *p* < 0.001) and have lower head circumferences (*OR* = 29.9; 95% CI 21.38–41.83; *p* < 0.001).

Low birth weight increased odds of abnormal Apgar at 5-min by 7 (*OR* = 7.05; 95% CI 3.86–23.88; *p* < 0.001) (Fig. 4A). The Apgar index is a method that assesses the newborn physical condition immediately after delivery by estimating five characteristics: heart rate, respiratory effort, muscle tone, reflex irritability, and skin colour, assigning a value of zero to two for each item [27]. The total value is the sum of the five components and a value of seven or more indicates that the child’s physical condition is good to excellent [27]. Scores calculated at 5 min post-partum can show normal (≥ 7) or abnormal (< 7) fetal-to-neonatal transition [28]. This study showed that 3% of newborns (75/2807) had low (< 7) Apgar at 5 min. Two previous studies showed the relationship between anaemia, low birth weight, low Apgar index, and stillbirth in Pakistan [46], and in a Canadian province [24].

Both *P*. *falciparum* and *P*. *vivax* malaria increase the risk of stillbirth, with the former responsible for 12–20% of stillbirths every year in sub-Saharan Africa [31]. Natural immunity acquired by pregnant women can potentially mask acute disease, leaving these women vulnerable to insidious outcomes including severe maternal anaemia and the death of the fetus before, during, or after delivery [8]. *Plasmodium vivax*, prevalent in Latin America, Southeast Asia, and Western Pacific, increases the risk of stillbirths by almost three times [8]. Here a stillbirth frequency of 0.8% (23/2807) was found, which is similar to that of 1.3% found in 2009 [18]. Figure 4B shows that mothers who presented perinatal fever were 12 times more likely to deliver a stillbirth (*OR* = 12.56; 95% CI 5.1–31.02; *p* < 0.001). Perinatal fever was both associated with maternal perinatal malaria (*OR* = 19.12; 95% CI 6.75–54.18; *p* < 0.001) and diagnosis of *P*. *falciparum* during pregnancy (*OR* = 2.9; 95% CI 1.39–6.05; *p* < 0.001) (Fig. 4B). Among these mothers, perinatal anaemia increased odds of stillbirths by 2.5 (*OR* = 2.5; 95% CI 1.02–6.42; *p* = 0.0444).

One fifth of the surveyed mothers (*n* = 564; 20.1%) did not undergo examination of parasites by light microscopy during pregnancy, thus missing the opportunity to diagnose asymptomatic infection. Among health professionals involved in prenatal care, malaria in pregnancy is still not a priority [33]. However, the National Malaria Control Program (PNCM) published a technical note in 2006 with recommendations to ensure that all pregnant women be tested for malaria in each prenatal visit to leverage early diagnosis and treatment in endemic areas in Brazil. In 2014, Stork Network Strategy (Rede Cegonha) reinforced the recommendations of the PNCM and emphasized that the examination of parasites by light microscopy in endemic regions should be performed during the six or more recommended prenatal visits [47]. Here, six or more prenatal visits decreased odds of low Apgar at 5-min by 59% (*OR* = 0.41; 95% CI 0.23–0.74; *p* = 0.003; Table 2) and stillbirth by 81% (*OR* = 0.19; 95% CI 0.04–0.93; *p* = 0.041; Table 3).

Malaria testing should occur in the antenatal care period because it is a determination from the federal government to every malarial-endemic municipality in the Brazilian Amazon [47]. Considering that pregnant women should be interested about their health statuses and the Hospital has malaria-testing exams available free-of-charge, it is reasonable to suppose that pregnant women (*n* = 564) who did not undergo malaria tests were not at risk of malaria exposure. If this is correct, the assumption they would get negative results if they had been tested is reasonable. Thus, pooling them together with those 2243 mothers who received malaria testing in the analysis can be justified (Table 1).

Here a multivariable regression model based on a stepwise variable selection method was applied. Although this approach has been widely used in public health studies and beyond, it suffers from a few issues [48]. A common and often neglected issue in multivariable regression is model overfitting due to the selection of covariates lacking mutual independence. With the inclusion of non-independent covariates in the final model, their respective regression coefficient values and the level of variance explained in the model (aka *R*-squared) can become biased to be high. With overfitting, a model can erroneously predict the magnitude or even direction of an association [48]. Alternatively, analyses of causation paths using DAGs with a SEM approach were undertaken (Fig. 4).

In the analysis of causation paths (Fig. 4), any parasite combination (*vivax* and/or *falciparum*) during pregnancy might lead to a newborn having low Apgar at 5-min (Model B1). An association between *P*. *falciparum* (single or mixed) in pregnancy and stillbirth was seen (Model B2). This association might be related to *P*. *falciparum* inducement of intrauterine subdevelopment [4].

## Limitations

This large-scale surveillance study adds important information about the web of causation contributing to maternal–fetal outcomes in pregnant women with malaria, however it does have some limitations. Nutritional status, alcoholism, or smoking—which should be important factors modulating anaemia in mothers—were not assessed. These factors are unobserved variables in the proposed DAGs (Fig. 1B). As 20.1% (*n* = 564) of mothers in this survey did not receive testing for malaria during pregnancy, it was assumed that if testing had been done, they would result negative. Missing data listed in Table 1 were not used in data analysis. In this analysis all continuous variables were categorized into binary variables, which leads to two drawbacks: one is loss of statistical power and the other is assuming a null effect between the continuous variable and the outcome within each binary category (no difference between a woman with haemoglobin = 7 g/dL and a woman with haemoglobin = 10.9 g/dL). The study design is a cross-sectional with pregnant women during labour or puerperal women up to 72 h after delivery. It was, therefore, assumed that exposures and antecedents of exposures preceded the outcomes (low Apgar at 5-min and stillbirth) at delivery.

## Conclusions

Here malaria risk factor on maternal–fetal health outcomes were assessed. Additive and independent effects only partially explained the underlying effects that maternal malaria can have on newborn outcomes. Causation of endemic malaria on the health of mothers and newborns is much more complex. The causal paths can be modulated by distal determinants in the causal framework. The conditions of mothers living environment tied to increased malaria risk can affect newborn outcomes after gestational period, including stillbirth.

## Supplementary Information

**Additional file 1.** “Research Tool” for clarification of the instrument used to obtain variables described in the manuscript from the study population.

**Additional file 2.** “Alternative results” for showing application of the same analytical approach as in Fig. 4 with mothers who did malaria testing (*n* = 2243).

## Data Availability

The dataset used and/or analysed during the current study are available from the corresponding author on reasonable request.
