# Supplementary material for: Causal effects on low Apgar at 5-min and stillbirth in a malaria maternal–fetal health outcome investigation: a large perinatal surveillance study in the Brazilian Amazon
Source: Malar J. 2021 Nov 25;20:444. doi: 10.1186/s12936-021-03981-y (PMC8614005; doi:10.1186/s12936-021-03981-y)
Supplement: Supplementary file 1 — Additional file 1. “Research Tool” for clarification of the instrument used to obtain variables described in the manuscript from the study population. [file 12936_2021_3981_MOESM1_ESM.docx]

*Additional file 1*

**Causal effects on low Apgar at 5-mins and stillbirth in a malaria maternal-fetal health outcome investigation: a large perinatal surveillance study in the Brazilian Amazon**

Julio Seijas Abel Chávez^1,2^, Melissa S. Nolan^3#^, Mary K. Lynn^3^, Maria José Francalino da Rocha^4^, Muana da Costa Araújo^5^, Fernando Luiz Affonso Fonseca^1^, Gabriel Zorello Laporta^1#^

**RESEARCH TOOL**

N° SURVEY: ........................

DATE OF SURVEY: ______/__ ___/________ RESEARCHER: _____________________________________________________________

| **1. PATIENT IDENTIFICATION** | | | | |
| --- | --- | --- | --- | --- |
| NAME | AGE (yrs.) | BIRTHDATE  (dd/mm/aa) | ADMISSION | |
|  |  |  | date | hours |
|  |  | / / | / / | : |

| **2. SOCIODEMOGRAPHIC CHARACTERISTICS** | | | | | | | | | |  |
| --- | --- | --- | --- | --- | --- | --- | --- | --- | --- | --- |
| HABITATION | | | EDUCATION | | | | MARITAL STATUS | | | |
| COUNTY | AREA | | ILLITERATE | SECONDARY | HIGH SCHOOL | COLLEGE | SINGLE | MARRIED | OTHER (divorced, widowed) | |
|  | URBAN | RURAL |  |  |  |  |  |  |  | |
|  |  |  |  |  |  |  |  |  |  |  |

| **3. OBSTETRIC DATA** | | | | | | | |  |
| --- | --- | --- | --- | --- | --- | --- | --- | --- |
| PARITY | | LMP  (dd/mm/aa) | GESTATIONAL AGE (weeks) | | | | N° CONSULTS DURING PREGNANCY | |
| N° DELIVERIES | N° ABORTION |  | from the LMP | <20 weeks | > 20 weeks | IGNORED |  |  |
|  |  | / / |  |  |  |  |  | |

| **4. CLINICAL DATA** | | | | | | | | | | | | | | |  |
| --- | --- | --- | --- | --- | --- | --- | --- | --- | --- | --- | --- | --- | --- | --- | --- |
| FEVER IN THE LAST WEEK (PRIOR TO THE DELIVERY) | | MALARIA CLINICAL HISTORY | | | | | | | TREATMENT DURING PREGNANCY | | MALARIA TESTING DURING PREGNANCY | | | | |
|  |  | PRIOR TO THE CURRENT PREGNANCY | | DURING THE CURRENT PREGNANCY | | | | |  |  |  |  |  |  |  |
|  |  |  |  | NO | YES | | | |  |  | NO | YES | | | |
| YES | NO | YES | NO |  | VIVAX | FALC | MIXED | Ignored | YES | NO |  | 1^st^ Trim. | 2^nd^ Trim. | 3^rd^ Trim. | |
|  |  |  |  |  |  |  |  |  |  |  |  |  |  |  | |

| **5. BEHAVIOURAL DATA** | | | | | | | | | | | |
| --- | --- | --- | --- | --- | --- | --- | --- | --- | --- | --- | --- |
| USE OF REPELLENT DURING PREGNANCY | | | | USE OF BEDNET DURING PREGNANCY | | | | USE OF AIR CONDITIONING DURING PREGNANCY | | | |
| NO | YES | | | NO | YES | | | NO | YES | | |
|  | 1^st^ Trim. | 2^nd^ Trim. | 3^rd^ Trim. |  | 1^st^ Trim. | 2^nd^ Trim. | 3^rd^ Trim. |  | 1^st^ Trim. | 2^nd^ Trim. | 3^rd^ Trim. |
|  |  |  |  |  |  |  |  |  |  |  |  |

| **6. DELIVERY DATA** | | | |
| --- | --- | --- | --- |
| DATE (dd/mm/aa) | HOUR (00:00) | TYPE | |
|  |  | VAGINAL BIRTH | CESAREAN BIRTH |
| / / | : |  |  |

| **7. NEONATAL DATA** | | | | | | | | |
| --- | --- | --- | --- | --- | --- | --- | --- | --- |
| WEIGHT (g) | APGAR | | Gestational age (weeks) | SEX | | Head circumference (cm) | Chest circumference (cm) | Height (cm) |
|  | 1’ | 5’ |  | male | female |  |  |  |
|  |  |  |  |  |  |  |  |  |

| **8. LABORATORIAL DATA** | | | | | | | | |  |
| --- | --- | --- | --- | --- | --- | --- | --- | --- | --- |
| HAEMOGLOBIN (g/dL) | BLOOD SMEAR TESTING FOR MALARIA BY LIGTH MICROSCOPY | | | | BLOOD SMEAR TESTING FOR MALARIA BY LIGTH MICROSCOPY | | | | |
|  | NEGATIVE | POSITIVE | | | NEGATIVO | POSITIVE | | | |
|  |  | VIVAX | FALC | MIXED |  | VIVAX | FALC | MIXED | |
|  |  |  |  |  |  |  |  |  | |
