# Supplementary material for: Causal effects on low Apgar at 5-min and stillbirth in a malaria maternal–fetal health outcome investigation: a large perinatal surveillance study in the Brazilian Amazon
Source: Malar J. 2021 Nov 25;20:444. doi: 10.1186/s12936-021-03981-y (PMC8614005; doi:10.1186/s12936-021-03981-y)
Supplement: Supplementary file 2 — Additional file 2. “Alternative results” for showing application of the same analytical approach as in Fig. 4 with mothers who did malaria testing (n = 2243). [file 12936_2021_3981_MOESM2_ESM.docx]

*Additional file 2*

**Causal effects on low Apgar at 5-mins and stillbirth in a malaria maternal-fetal health outcome investigation: a large perinatal surveillance study in the Brazilian Amazon**

Julio Seijas Abel Chávez^1,2^, Melissa S. Nolan^3#^, Mary K. Lynn^3^, Maria José Francalino da Rocha^4^, Muana da Costa Araújo^5^, Fernando Luiz Affonso Fonseca^1^, Gabriel Zorello Laporta^1#^

^
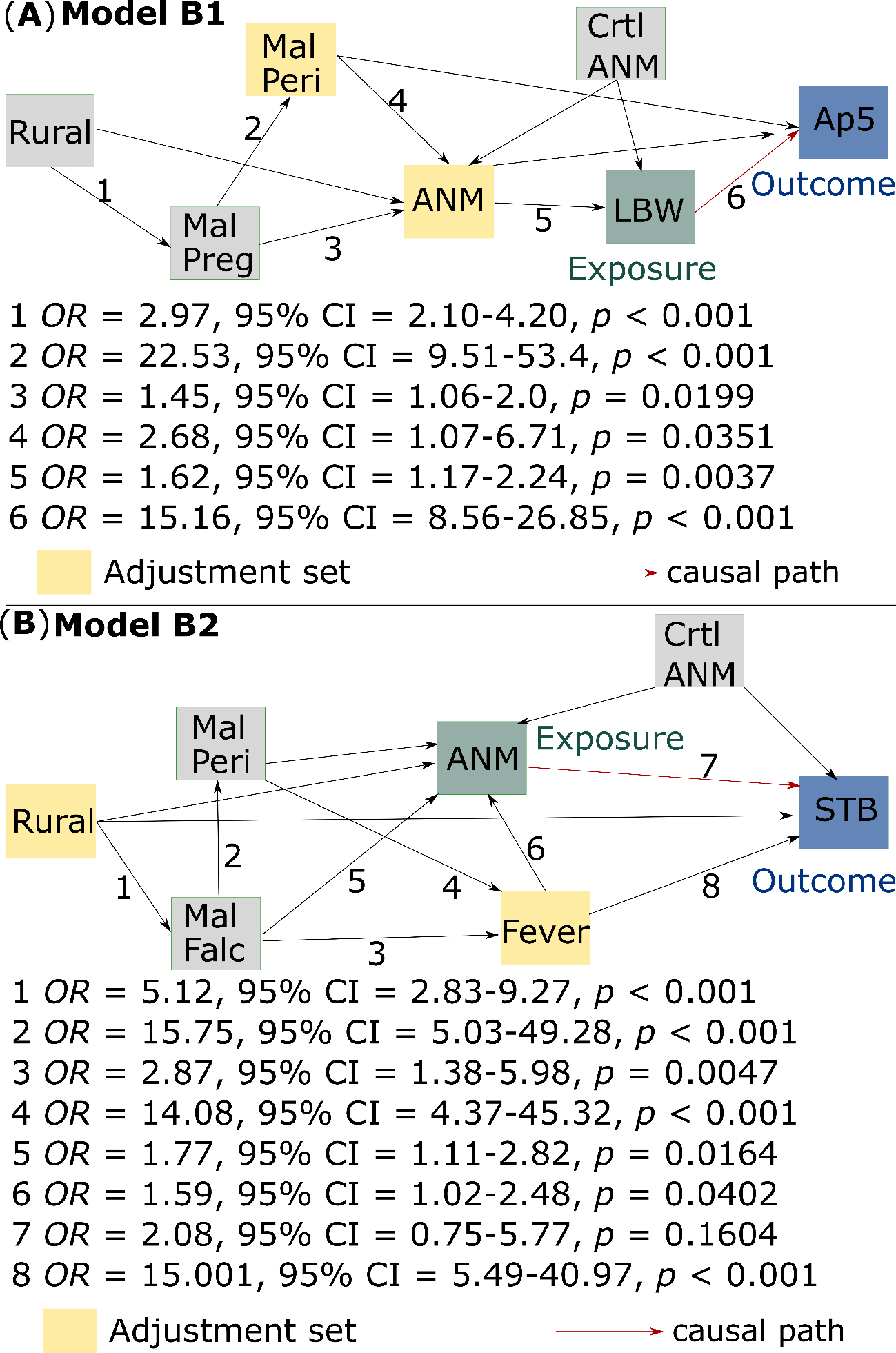
^
